# Associations of Short Sleep and Shift Work Status with Hypertension among Black and White Americans

**DOI:** 10.1155/2015/697275

**Published:** 2015-10-01

**Authors:** Mirnova E. Ceïde, Abhishek Pandey, Joe Ravenell, Margaret Donat, Gbenga Ogedegbe, Girardin Jean-Louis

**Affiliations:** ^1^Montefiore Medical Center, Department of Psychiatry and Behavioral Science, 111 East 210 Street, Bronx, NY 10467, USA; ^2^Brooklyn Health Disparities Center, Department of Medicine, SUNY Downstate Medical Center, Brooklyn, NY 11203, USA; ^3^Center for Healthful Behavior Change, Department of Population Health, NYU Langone Medical Center, New York, NY 10016, USA

## Abstract

*Objective*. The purpose of this study was to investigate whether short sleepers (<6 hrs) who worked the non-day-shift were at greater likelihood of reporting hypertension and if these associations varied by individuals' ethnicity. *Methods*. Analysis was based on the 2010 National Health Interview Survey (NHIS). A total of 59,199 American adults provided valid data for the present analyses (mean age = 46.2 ± 17.7 years; 51.5% were female). Respondents provided work schedule and estimated habitual sleep durations as well as self-report of chronic conditions. *Results*. Of the sample, 30.8% reported a diagnosis of hypertension, 79.1% reported daytime shift work, 11.0% reported rotating shift work, and 4.0% reported night shift work. Logistic regression analysis showed that shift work was significantly associated with hypertension among Blacks [OR = 1.35, CI: 1.06–1.72. *P* < 0.05], but not among Whites [OR = 1.01, CI: 0.85–1.20, NS]. Black shift workers sleeping less than 6 hours had significantly increased odds of reporting hypertension [OR = 1.81, CI: 1.29–2.54, *P* < 0.01], while their White counterparts did not [OR = 1.17, CI: 0.90–1.52, NS]. *Conclusions*. Findings suggest that Black Americans working the non-day-shift especially with short sleep duration have increased odds of reporting hypertension.

## 1. Introduction

Over the past thirty years, various hazards have been associated with working outside the conventional day shift, which is commonly referred to as shift work. Shift work has been linked to gastrointestinal disease [1], metabolic syndrome [2–4], cardiovascular disease [5–10], and cancer [11, 12]. Evidence shows that shift work has detrimental effects on mood, cognitive performance, and family life [13–15]. Additionally, recent studies suggest that circadian disruption caused by shift work may result in impaired glucose metabolism, type II diabetes, and hypertension [4, 16, 17].

Generally, shift workers are at greater risk for cardiovascular disease including obesity [18, 19], diabetes [4, 16, 20], hypertension [17, 21–25], and metabolic syndrome [2, 3, 26–29]. A 10-year follow-up study of Japanese factory workers found that those who were mainly shift workers had higher body mass index (BMI) and total cholesterol than their day shift counterparts [19]. In addition, individuals who switched from day to shift work had on average 1.03 kg/m^2^ increased BMI [19]. A similar 14-year cohort study of Japanese male steel workers revealed that rotating shift work was an independent risk factor for increased systolic and diastolic blood pressure [25]. These results are consistent with a prospective cohort study of British workers who were studied over a period of 45 years [29]. Investigators demonstrated that males working the night/early morning shifts tended to have less favorable outcome for waist circumference, body mass index, triglycerides, HDL-C, and Hemoglobin A1C; however, systolic and diastolic blood pressure and total cholesterol were unaffected. By contrast, female workers mainly showed elevated triglyceride levels [29]. It is of interest to ascertain whether these associations are independent of the amount of habitual sleep duration, as shift work is known to result in shorter sleep duration (<6.5 hrs) [30], which is also linked to cardiovascular disease [31, 32].

Relatively little is known concerning ethnicity and its association with shift work and hypertension, an important cardiovascular disease. It is important to examine ethnic effects on such associations because of data suggesting that Black short sleepers may have an increased risk of hypertension [8]. Furthermore, data from the Nurses' Health Study II suggested that Black women on rotating shift schedules had a 46% increased risk of developing hypertension compared with their White counterparts [17]. The purpose of this study was to investigate whether short sleepers (<6 hrs) working the nonday shift were at greater risk of reporting hypertension. We also explored whether associations varied based on individuals' ethnicity.

## 2. Methods

### 2.1. Participants

A total of 59,199 Americans (age range: 18–85 years) who participated in the 2010 National Health Interview Survey (NHIS) provided sociodemographic and subjective data as well as data on self-reported chronic conditions for the present analysis. Analysis focused on associations among shift work, short sleep, and hypertension, while examining effects of ethnicity on these associations. Final weights provided by the CDC were applied to all analyses to adjust for the use of complex design in the NHIS. Of the sample, 82.8% were of White ethnicity and 17.2% were of Black ethnicity. Adults of both sexes were represented; 48.5% of the volunteers were men and 51.5% were women.

### 2.2. Procedures

NHIS is an ongoing, cross-sectional, in-person household interview survey conducted annually by the National Center for Health Statistics of the Centers for Disease Control and Prevention. The NHIS uses a multistage area probability design sampling noninstitutionalized representative of US civilian population. Probability samples of the adult population of all 50 states and the District of Columbia were obtained. The final sample was characterized by a response rate of 90%. Details on sample design can be found in Design and Estimation for the National Health Interview Survey [33].

During face-to-face interviews conducted by trained interviewers from the US Census Bureau, volunteers provided sociodemographic data and information about self-reported chronic conditions. The chronic conditions included hypertension and diabetes. Ethnicity was assessed by the standards recommended by Interagency Committee for the Review of the Racial and Ethnic Standards. Participants responded to the question “which one of these groups would you say BEST represents your race?”

Health risk data included smoking status and alcohol intake. Smoking status was defined as current, former, or never. Alcohol intake was assessed based on the responses to the following questions: “in your entire life, have you had at least 12 drinks of any type of alcoholic beverage?” Respondents who consumed <12 drinks in their entire life were classified as never-drinkers; those who consumed ≥12 drinks in any year or their entire life were classified as drinkers.

Self-reported diseases were defined based on affirmative responses to the following questions: “have you ever been told by a doctor or other health professional that you have [disease or condition]?” Thus, hypertension was defined by an affirmative response to the question: “have you ever been told by a doctor or other health professionals that you had hypertension?” Diabetes was defined by an affirmative response to the question: “other than during pregnancy, have you ever been told that you have diabetes?” Participants also estimated habitual sleep duration (using full hour units, i.e., 5 hours, 6 hours, and 7 hours); no information on specific sleep disorders was elicited during the interview. Habitual short sleep duration was coded as (<6 hours/night), which was referenced to 7-8 hours/night sleepers. Short sleep duration was defined as <6 hrs because this is consistent with a number of previous studies evaluating negative outcomes of short sleep [20, 31, 34–37]. Long sleep duration (>9 hrs) was not included in this study as it is less frequent than short sleep duration among shift workers [32, 35, 38]. Participants were also asked to rate their mood within the last 30 days prior to the interview. Using mood indices (e.g., feeling of sadness, hopelessness, worthlessness, and poor effort), a depression severity score was generated which was a composite score estimated using the K-6 scaling system [39]. Responses were used to generate a score ranging from 0 to 24. Scores ≥13 indicated a greater degree of emotional distress [40].

Surveys were conducted using computer-assisted personal interviewing (CAPI), which utilizes a computer program for data collection that guides the interviewer through the questionnaire. The interviewer enters survey responses directly into the computer. The program determines through a computer algorithm whether data entered by the user match against all possible responses to specific questions; the program also checks for consistency against other data collected during the interview and saves the responses into a survey data file [41].

### 2.3. Statistical Analysis

Since the NHIS dataset includes data from different samples using a multistage area probability sampling design, all analyses performed in this study were based on weighted statistics using the weights provided with the NHIS dataset. These final weights that accompany the dataset represent a product of weights for corresponding units computed in each of the sampling stages to account for variations in sampling strategies that might affect generalization of final results [41].

Frequency and measures of central tendency were used to describe the sample. In preliminary analyses, Pearson and Spearman correlations were used to explore relationships between variables of interest; only factors showing a *P* value <0.05 were considered in the final regression model [40]. ANOVA was used for group mean comparisons, and Chi square test was employed to assess differences in categorical variables.

Using multivariate-adjusted logistic regression analyses, we examined associations of shift work (evening, night, or rotating shift work schedules) and short sleep duration with hypertension stratified by ethnicity; stratified analysis was justified by preliminary analysis showing significant interaction between ethnicity and shift work status (Wald = 40.26; *P* < 0.01) and short sleep (Wald = 43.34; *P* < 0.01), even with adjustment for covariates. The first model assessed odds of reporting hypertension among shift workers. The second model determined odds of reporting hypertension among shift workers who were also short sleepers. Covariates entered in the models were gender, age, income, education, tobacco use, alcohol use, emotional distress, and diabetes. BMI was not included as a covariate in the final models, as it was not statistically significant in preliminary univariate analyses. All analyses were performed using SPSS 20.0.

## 3. Results

Of the sample, 30.8% reported a diagnosis of hypertension, 79.1% reported daytime shift work, 5.9% reported evening shift, 11.0% reported rotating shift, and 4.0 % reported night shift work.  Table 1 illustrates the demographic and comorbid characteristics of both White and Black participants. Blacks were more likely to report hypertension compared with their White counterparts (37.9% versus 29.7%).  Table 2 illustrates the distribution of work schedules among White and Black participants. Of note, a higher percentage of Blacks worked the night shift (5.9% versus 3.2%) or rotating shift (13.2% versus 9.5%) schedules relative to their White counterparts.

In Table 3, logistic regression analysis shows that shift work was significantly associated with hypertension among Black shift workers, but not among White shift workers. Among White shift workers, age, tobacco use, and diabetes were significantly associated with increased odds of reporting hypertension. Among Black shift workers, male gender, age, alcohol use, and diabetes were associated with increased odds of reporting hypertension.

Table 4 shows results of logistic regression analysis of shift workers who were also classified as short sleepers (<6 hrs), referenced to those sleeping 7-8 hours. Analysis showed that Black shift workers classified as short sleepers had significantly increased odds of reporting hypertension. Analysis showed no significant increases in odds of reporting hypertension among White shift workers.

## 4. Discussion

The goal of this study was to evaluate whether shift workers who also experience short sleep duration are more likely to report hypertension among Black and White Americans. Our study showed that shift work was only significantly associated with increased odds of reporting hypertension among Black participants, but not among White shift workers. In addition, Black shift workers, reporting short sleep duration, had increased odds of reporting hypertension compared with Black shift workers with healthy sleep duration (7-8 hours). Of interest, these associations were not significant for White participants.

The lack of significant finding among White participants is inconsistent with previous findings especially in the context of European studies, which tend to show increased cardiovascular risk among White shift workers [3, 5, 6, 26, 29, 42–44]. We should note, however, that our findings are consistent with more recent studies regarding increased odds of hypertension among Black shift workers as opposed to White shift workers [17]. These discrepancies could not be explained by differences in sociodemographic and health risk characteristics on the basis of individuals' ethnicity. Logistic regression indicated that age, male gender, and diabetes were all significant contributors to increased odds of reporting hypertension among both White and Black participants. Tobacco use was a significant contributor to increased odds of hypertension in the White participants, and alcohol use was a significant contributor in Black participants. The fact that diabetes was the strongest predictor in our model accords with previous findings suggesting that nonconventional shift work increases the risk of hypertension and diabetes. Indeed, a recent laboratory study, which mimicked the conditions of shift work by combining sleep restriction and circadian rhythm disturbance, resulted in increased postprandial glucose and decreased resting metabolic rate [16]. These findings are consistent with a study of nurses which found a dose dependent relationship between years working on rotating shift and risk of diabetes [4].

Our findings and previous literature suggest a relationship between shift work and short sleep duration with the presence of hypertension. Still a mechanism linking short sleep duration and shift work to hypertension is lacking. One hypothesis postulates that short sleep duration leads to sympathetic activation, which in turn results in high blood pressure [45, 46]. Other theories propose a disturbance in the circadian rhythm as the catalyst for a variety of pathways that lead to hypertension [23, 47–49]. One such study noted that people who work rotating or night shift work tended to display impaired blood pressure dipping at night after just one night shift [50]. Others propose that disturbances in circadian rhythm may lead to impaired endothelial function perhaps via decreased nitric oxide [50] and/or myocyte hypertrophy and fibrosis in animal studies [51]. In healthy men, cortisol secretion is inhibited during the first 4 hours of sleep [52]. A study of textile factory workers found that hair cortisol levels and BMI were increased in shift workers [53]. Disturbances in the circadian rhythm related transcription factor CLOCK affect acetylation of glucocorticoid receptors resulting in increased translation of glucocorticoid receptors and subsequent effects on end organ systems [48]. It is likely that the combination of sympathetic activation, endothelial dysfunction, and increased cortisol activity all contribute to the development of cardiovascular disease in shift workers especially those with short sleep duration.

Our study has notable limitations. First, we relied on subjective report of hypertension, especially diagnosis of hypertension in the past, which could not be verified with objective data. Secondly, we did not have data on subjective sleep disturbance or report of insomnia. Based on the Penn State cohort, those with insomnia and short sleep duration have a higher incidence of hypertension. Poor sleepers without insomnia had a marginal increased hypertension after adjusting for obesity [54]. Also in the Penn State cohort nonobese people, with subjective sleep disturbance, had an increased incidence of obesity [55]. Thirdly, night shift workers and rotating shift workers were categorized as a singular non-day-shift group. Previous work has shown that permanent night shift workers may be exposed to more sleep deprivation and may have different health behaviors like tobacco use, which would affect the risk of hypertension [56, 57]. Fourth, important information was unavailable such as presence of antihypertensive medications and diagnosis of sleep apnea. Additionally, we utilized cross-sectional data; thus, we could not ascertain long-term effects of shift work on hypertension or whether incidence of hypertension would be greater among shift workers. A previous cohort study of Belgian workers found an increased incidence of metabolic syndrome as well as a dose-dependent relationship [27]. Likewise, we could not establish the mechanism by which short sleep duration and shift work influenced hypertension.

Notwithstanding the limitations described above, our study has several strengths. First, we used a population-based representative sample of US adults, which enhanced generalizability of our findings. Many studies in the past have been conducted in relatively homogenous populations in Scandinavia [3, 58, 59] or Asia [22, 23, 25, 60]. Second, we investigated the association between ethnicity and short sleep and hypertension among shift workers, which heretofore has not been undertaken. Future studies should explore those associations among Hispanic and Asian populations as well. Investigations should also include large prospective cohort studies in the US utilizing diverse populations to provide information on incidence of hypertension in shift workers with short sleep duration.

## 5. Conclusion

Minimizing health risks among shift workers is a daunting challenge, a fact that has captured the attention of national organizations like the Center for Disease Control and Prevention. Our study suggests the need to explore modifiable factors that may compound detrimental effects of shift work and short sleep including alcohol consumption and tobacco use, which are established risk factors for cardiovascular disease among shift workers [42, 61, 62]. One promising intervention in this area is the implementation of workplace smoking cessation programs, which may reduce cardiovascular risk among shift workers. Another target of intervention may involve increased opportunity for healthy sleep duration by maintaining individuals on rotating shifts rather than on permanent night shift, which could mitigate risk of hypertension in shift workers [63, 64]. Improving availability of health food options in the workplace and providing opportunities for physical activity may also prove beneficial, as shift workers tend to eat more energy-dense foods and have less opportunity for physical activity [65]. In sum, a particular focus on reducing identified risk factors among Black short sleepers is of utmost importance since they are more vulnerable to the cardiovascular hazards of shift work.

## Figures and Tables

**Table 1:** Baseline data of participants in the 2010 National Health Interview Survey (NHIS).

Sociodemographic, health risk, and medical characteristics of NHIS participants						
Variable	Whites (SE)	95% (CI) lower	95% (CI) upper	Blacks (SE)	95% (CI) lower	95% (CI) upper
Age (mean)	46.9 (0.2)	46.6	47.4	43.2 (0.4)	42.5	43.9
Female gender (%)	51.0 (0.4)	50.2	51.9	54.8 (0.9)	53.1	56.6
Completed high school (%)	87.7 (0.3)	87.0	88.3	85.0 (0.6)	83.7	86.2
Income > 35 K (%)	68.0 (0.5)	66.9	69.0	50 (1.1)	47.8	52.3
Ever smoked 100 cigs in life (%)	43.0 (0.5)	42.1	43.9	34.4 (0.9)	32.6	36.2
Current drinker (%)	81.8 (0.4)	81.0	82.6	70.4 (0.9)	68.5	72.1
Emotional distress (%)	2.7 (0.1)	2.5	3.0	3.2 (0.3)	2.7	3.9
Diabetes (%)	9.9 (0.2)	9.4	10.4	13.2 (0.5)	12.2	14.2
Hypertension (%)	29.7 (0.4)	29.1	30.5	37.9 (1)	36.1	39.8

SE:  standard error, CI:  confidence interval.

**Table 2:** Distribution of work schedules among white and black NHIS participants.

Work schedules of white and black participants in the 2010 NHIS data						
Variable	Whites (SE)	95% (CI) lower	95% (CI) upper	Blacks (SE)	95% (CI) lower	95% (CI) upper
Regular daytime shift (%)	72.1 (0.5)	71.0	73.2	65.7 (1.2)	63.3	67.9
Regular evening shift (%)	4.9 (0.2)	4.5	5.5	8.0 (0.7)	6.7	9.6
Regular night shift (%)	3.2 (0.2)	2.9	3.6	5.9 (0.6)	4.8	7.1
Rotating shift (%)	9.5 (0.3)	8.8	10.2	13.2 (0.8)	11.7	14.8

SE: standard error, CI: confidence interval.

**Table 3:** Logistic regression analysis showing adjusted odds ratios (OR) and confidence intervals (CI) for hypertension among white (top pane) and black (bottom pane) shift workers.

Likelihood of reporting hypertension among white and black shift workers				
Variable	OR	95% CI lower	95% CI upper	*P*
White shift worker	1.01	0.85	1.20	0.88
Gender	0.83	0.75	0.93	<0.01
Age	1.06	1.06	1.07	<0.01
Income	0.97	0.84	1.12	0.65
Tobacco use	1.26	1.11	1.43	<0.01
Alcohol use	1.26	1.06	1.50	<0.01
Emotional distress	1.67	1.05	2.67	<0.05
Diabetes	3.74	3.10	4.52	<0.01

Variable	OR	95% CI Lower	95% CI Upper	*P*

Black shift worker	1.35	1.06	1.72	<0.05
Gender	1.23	0.97	1.56	0.09
Age	1.08	1.07	1.09	<0.01
Income	0.85	0.65	1.11	0.23
Tobacco use	1.24	0.96	1.60	0.10
Alcohol use	1.48	1.13	1.94	<0.01
Emotional distress	3.07	1.56	6.05	<0.01
Diabetes	6.28	3.99	9.88	<0.01

**Table 4:** Logistic regression analysis indicating adjusted odds ratios (OR) and confidence intervals (CI) for hypertension among white (top pane) and black (bottom pane) shift workers reporting short sleep duration.

Likelihood of reporting hypertension among white and black shift workers reporting short sleep				
Variable	OR	95% CI lower	95% CI upper	*P*
White shift worker with short sleep	1.17	0.90	1.52	0.23
Gender	0.79	0.69	0.91	<0.01
Age	1.06	1.06	1.07	<0.01
Income	0.93	0.77	1.11	0.41
Tobacco use	1.03	1.01	1.05	<0.01
Alcohol use	1.19	0.93	1.52	0.16
Emotional distress	1.52	0.87	2.67	0.14
Diabetes	3.58	2.74	4.67	<0.01

Variable	OR	95% CI lower	95% CI upper	*P*

Black shift worker with short sleep	1.81	1.29	2.54	<0.01
Gender	1.40	1.01	1.94	<0.05
Age	1.09	1.08	1.11	<0.01
Income	0.91	0.64	1.29	0.59
Tobacco use	1.26	0.91	1.75	0.17
Alcohol use	1.79	1.17	2.75	<0.01
Emotional distress	3.55	0.85	14.77	0.08
Diabetes	5.93	3.16	11.11	<0.01
